# Hand-Writing Motion Tracking with Vision-Inertial Sensor Fusion: Calibration and Error Correction

**DOI:** 10.3390/s140915641

**Published:** 2014-08-25

**Authors:** Shengli Zhou, Fei Fei, Guanglie Zhang, Yunhui Liu, Wen J. Li

**Affiliations:** 1 School of Astronautics, Northwestern Polytechnical University, Xi'an 710000, China; E-Mail: shengli.zhou@foxmail.com; 2 Department of Mechanical and Biomedical Engineering, City University of Hong Kong, Hong Kong, China; E-Mails: feifei@cityu.edu.hk (F.F.); gl.zhang@cityu.edu.hk (G.Z.); 3 Department of Mechanical and Automation Engineering, The Chinese University of Hong Kong, Hong Kong, China; E-Mail: yhliu@mae.cuhk.edu.hk

**Keywords:** MEMS-based motion tracking, vision-based motion tracking, inertial sensor calibration, stochastic error modeling, sensors fusion, human motion tracking

## Abstract

The purpose of this study was to improve the accuracy of real-time ego-motion tracking through inertial sensor and vision sensor fusion. Due to low sampling rates supported by web-based vision sensor and accumulation of errors in inertial sensors, ego-motion tracking with vision sensors is commonly afflicted by slow updating rates, while motion tracking with inertial sensor suffers from rapid deterioration in accuracy with time. This paper starts with a discussion of developed algorithms for calibrating two relative rotations of the system using only one reference image. Next, stochastic noises associated with the inertial sensor are identified using Allan Variance analysis, and modeled according to their characteristics. Finally, the proposed models are incorporated into an extended Kalman filter for inertial sensor and vision sensor fusion. Compared with results from conventional sensor fusion models, we have shown that ego-motion tracking can be greatly enhanced using the proposed error correction model.

## 1. Introduction

Motion tracking technologies which aim at translating human motion into computer-understandable instructions have drawn much attention in the past decade. Potential applications in human-to-machine interface technologies include controlling mobile and flying robots, and navigation and control in augmented reality environments. In general, current motion tracking technologies derive pose estimates from electrical signals received from mechanical, magnetic, acoustic, inertial, optical, radio or microwave sensors [1–3]. When applied to gesture recognition, most of these technologies can be used alone with good results, but when it comes to ego-motion tracking (3D motion tracking within an environment using a camera), none of these technologies is good enough on its own owing to limitations in volume, mobility, accuracy, latency, or tracking range.

For example, microelectromechanical systems (MEMS)-based inertial sensors are lightweight, and good for fast motion tracking, but they lack long term stability due to the problem of severe zero drift [4]. Vision sensors may be more accurate in tracking the motion with little accumulation of errors [5], but due to motion blur their ability to resolve fast movements is poor. They also suffer from line-of-sight limitations, and have problems in distinguishing between rotational and translational motion [6]. To overcome the inherent shortcomings of a single sensor, we present results from our custom-built system integrating a MEMS-based inertial sensor (consisting of accelerometers and gyroscopes, and is sometimes refer to as a “μIMU”) and a web-based camera (*i.e.*, a vision sensor) for motion tracking. For convenience of reference, we will call this μIMU + Camera system the “μIC system” from here on. The MEMS inertial sensor which consists of a 3-axis accelerometer and three single axis gyroscopes is able to measure the acceleration and angular rate, while the vision sensor is able to estimate its pose (position and orientation) relative to a checkerboard (used as a visual landmark). By fusing these two sensors, the drift problem of inertial sensor can be corrected when visual measurements are available. In the interval between two visual measurements, or when the images are blurred or lost due to fast movements, the poses are estimated by inertial measurement. Based on this sensor fusion concept, we have demonstrated a more accurate and reliable motion tracking system, which is described in detail in this paper.

Although significant work has been done in the past few years on inertial sensors as well as vision sensors, the majority of the reported work has focused on one or more of the following aspects: (1) relative pose calibration between inertial sensor and vision sensor [7,8]; (2) deterministic errors calibration of inertial sensor [9,10]; (3) stochastic errors identification [11–15], and modeling of inertial sensor (the majority of these work seek to model the stochastic errors in gyroscopes [12,16]); and (4) algorithms of inertial sensor and vision sensor fusion [17–20]. There are relatively few papers addressing the systematic calibration of a μIC system, which incorporates the orientation calibration of checkerboard and gravitational force as well as the relative calibration of inertial sensor and vision sensor. There are also only a few papers discussing how to combine stochastic error identification and modeling with inertial sensor and vision sensor fusion. This paper covers the following aspects: (1) systematic calibration for a μIC system; (2) deterministic and stochastic error identification and error modeling; (3) real-time pose estimation and correction; and (4) temporal information retrieval by using dynamic time warping (DTW).

The remainder of the paper is organized as follows: Section 2 presents the system setup, sensors and system calibrations, stochastic errors analysis by using Allan variance, and error modeling of the stochastic errors. The experimental results are discussed in Section 3. Finally, the conclusions are presented in Section 4.

## 2. Methodologies of Motion Tracking with μIC System

### 2.1. System Setup

**Figure 1.**
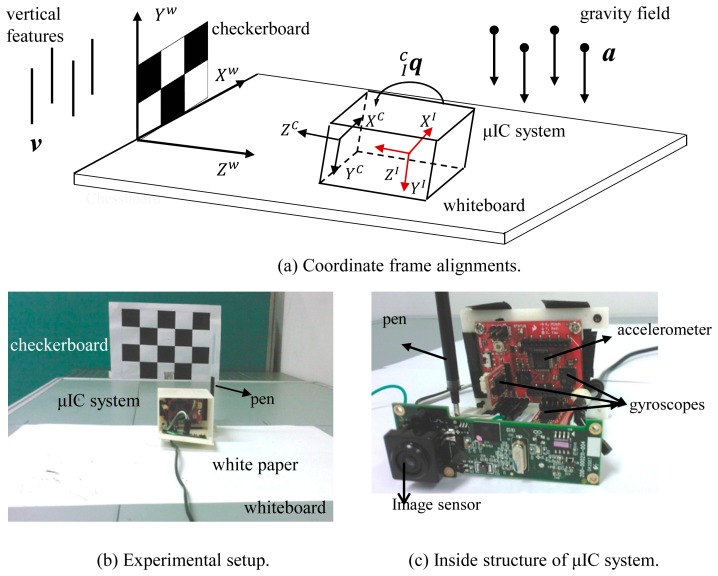
(**a**) Coordinate frame alignment; (**b**) Real experimental setup; (**c**) Inside structure of μIC system.

As shown in Figure 1, the experimental setup consists of a 3 × 4 checkerboard pattern, and a custom-built μIC system. The μIC system contains three single axis MEMS gyroscopes (LISY300AL gyroscope, STMicroelectronics, Geneva, Switzerland), a tri-axial MEMS accelerometer (MMA7260 accelerometer, Freescale, Austin, TX, USA), and a web-based vision sensor (Logitech QuickCam Pro 9000, Logitech, Inc., Fremont, CA, USA). With the accelerometers and gyroscope, the accelerations and angular rates of the devices can be measured, which can then be integrated into position and orientation. The checkerboard here is used by vision sensor for positioning. Knowing world coordinates of the corners on the checkerboard and their corresponding image coordinates, it is able to estimate the relative position of camera w.r.t. the world coordinate system. Consequently, the measurements from these two sensors can be fused together.

The maximum sampling rate of the vision sensor is 30 fps, but only about 5 fps has been utilized in this work. The μIMU and vision sensor are fixed in a box, so their relative position will not be changed during the experiments. A pen is attached to one side of the μIC system (Figure 1b), so the trajectory of the movement can be recorded on a normal white paper.

### 2.2. Inertial Sensor Calibration

Different from motion recognition, the accuracy of motion tracking is highly dependent on the accuracy of the exact angular rates and accelerations. Given the initial orientation and position of the device, the relative positions of the inertial sensor can be obtained in two steps. First, the orientation is updated using the angular rates measured by gyroscope. Second, the accelerations are rotated to the world coordinate frame by using the updated orientation. After the gravitational acceleration, g, is compensated for, the positions can be determined by double integrating the resultant accelerations. However, since the MEMS sensors inherently suffer from high random noise and time varying bias, the performance of the tracking system may degrade if the variants in accelerometer and gyroscope noises are not modeled and compensated properly [12]. This means that the inertial sensor needs to be calibrated carefully. There are two types of error sources in an inertial sensor [21]: deterministic errors, and stochastic errors. Deterministic errors include constant biases, scale factors and non-orthogonalities. These can be directly removed from measurements. Stochastic errors include angle/velocity walk, bias instability, rate random walk, rate ramp, and so on [11], which cannot be directly removed from the measurements, and should be modeled as stochastic process.

#### 2.2.1. Deterministic Error Determination

The mathematical model describing the accelerometer measurements and calibrated accelerations can be expressed as follows [10]:

(1) $$\begin{array}{lll} \left[\begin{array}{l} a_{x}\\ a_{y}\\ a_{z} \end{array}\right]= & \left[\begin{array}{lll} m_{xx} & m_{xy} & m_{xz}\\ m_{xy} & m_{yy} & m_{yz}\\ m_{xz} & m_{yz} & m_{zz} \end{array}\right] & \left[\begin{array}{l} a_{x}^{\prime }-b_{x}\\ a_{y}^{\prime }-b_{y}\\ a_{z}^{\prime }-b_{z} \end{array}\right] \end{array}$$

where *b_x_*, *b_y_*, and *b_z_* represent constant biases along each axis; $a_{x}^{\prime }$, $a_{y}^{\prime }$, *and* $a_{z}^{\prime }$ represent measured accelerations by accelerometer when it is in static condition; the diagonal elements of ***m*** matrix represent scale factors, and the off-diagonal elements represent non-orthogonalities. The basic principle for estimating the ***m*** matrix and the bias vector is that the modulus of modeled accelerations equals gravitational acceleration when the device is in a static condition. Defining the calibration error *r*(***x***) is defined as the difference between the squared sum of modeled accelerations and the squared gravitational acceleration,

(2) $$r(\text{x})=a_{x}^{2}+a_{y}^{2}+a_{z}^{2}-g^{2}$$

where ***x*** = (*m_xx_*, *m_xy_*, … , *b_z_*)*^T^*. The inertial sensor calibration reduces to finding the vector ***x*** that could minimize the calibration error. Since there are nine unknowns in Equation (2), nine sets of measurements are required to solve this nonlinear problem. These nine sets are collected by making the accelerometer statically point towards nine different directions.

There are many algorithms that could be used to find the solution to the nonlinear least square problem. For example, Newton's method, secant method, Gauss-Newton's method and so on. Among them, Newton's method requires the calculation of second order derivatives. Considering there are 9 unknowns in *r(x)*, 45 s order derivatives should be calculated, which is a huge task. Besides, Newton's method may converge to maximum or saddle point as well as minimum. Though secant method does not require derivative calculation, its convergence rate is slower than Newton's method. If there is a point where the first derivative equals zero on the interval between initial point and optimal point, the algorithm may not converge at all. Compared with them, Gauss-Newton's method only requires first order derivative calculation, and it is much easy to implement. So Gauss-Newton's method [10] is applied to iteratively optimize the estimation in the direction:

(3) $$d_{k}={\left(J^{T}J\right)}^{-1}(J^{T}(-r))$$

where *r* represents the calibration error vector; *J* represents the Jacobian of calibration error, which is calculated by:

(4) $$J(x)=\frac{\partial r}{\partial x}=\left[\begin{array}{ccc} \frac{\partial r_{1}}{\partial m_{xx}} & \cdots & \frac{\partial r_{1}}{\partial b_{z}}\\ \vdots & \ddots & \vdots \\ \frac{\partial r_{9}}{\partial m_{xx}} & \cdots & \frac{\partial r_{9}}{\partial b_{z}} \end{array}\right]$$

**Figure 2.**
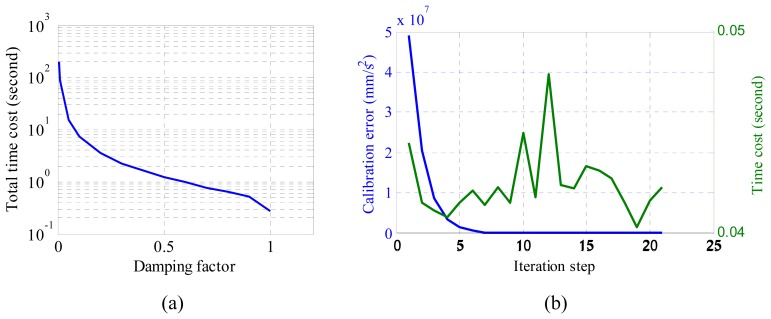
(**a**) Total computational time cost w.r.t. damping factor by using the Gaussian-Newton's method; (**b**) Calibration error and time cost of each iteration *versus* iteration step when damping factor is 0.6.

Next, the solution to the least square problem is updated as:

(5) $$x_{k+1}=x_{k}+\alpha_{k}d_{k}$$

where *α_k_* is the damping factor that controls the converging speed of the algorithm. If the damping factor is big, the algorithm will converge very fast. Otherwise, it may converge slowly. The total time cost w.r.t. different damping factors is plotted in Figure 2a. From this figure, we find the total time required for calibration drops sharply with the increase if damping factor.

Gauss-Newton's method is also very sensitive to initial approximation. If the initial approximate solution is near to the correct one, the algorithm will converge to the correct solution very fast. However, if the current solution is far from the correct one or the linear system is ill-conditioned, the algorithm will converge very slowly or even fail to converge [22]. Hence, the initial guess needs to be set carefully. If the accelerometer had been fabricated perfectly, the scale factors should be one and the non-orthogonalities should be zero, so the initial guess of *m* matrix is assumed to be an identity matrix. For initial bias estimation, due to the existence of gravitational acceleration, it is not trivial to get an estimation that is close to being true from a single measurement. We adopted the *six-position* method [23]. The method requires the inertial sensor to be placed on a level surface with the sensitive axis pointing alternatively up and down, so the estimated bias of the sensitive axis is:

(6) $$b_{i}=\frac{a_{i}^{\text{up}}+a_{i}^{\text{down}}}{2}$$

where $a_{i}^{\text{up}}$ and $a_{i}^{\text{down}}$ represent the measurements of the sensitive axis when it is pointing upwards or downwards. Next starting with this initial estimation of the parameters, the Gauss-Newton's algorithm refines the estimates until the maximum iteration value is reached, or the criterion for stopping the iteration is satisfied:

(7) |r(m,b)|<ε

where *ε* is a threshold, and is set to 1.00 mm^2^/s^4^ after several calibrations. The calibration error and the time cost in each step by using the Gauss-Newton's method are plotted in Figure 2b. We can see from the figure that the calibration error is decreasing with the increase of running steps. After running about 22 steps, the required precision has been reached.

#### 2.2.2. Stochastic Error Determination

**Figure 3.**
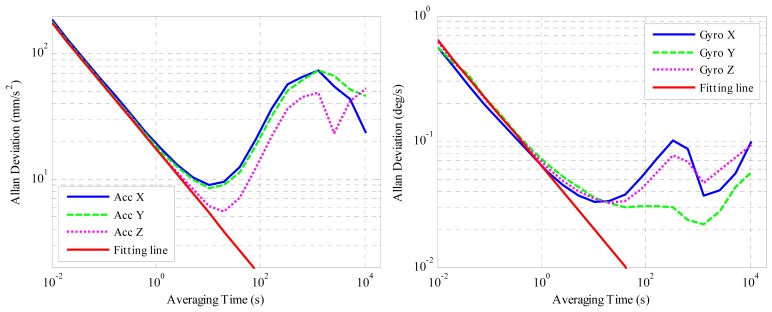
(**a**) Allan deviation plot for the accelerometer; (**b**) Allan deviation plot for the gyroscope.

Several approaches are available for stochastic error identification of an inertial sensor, e.g.: (1) Autocorrelation function approach [13], which is not practical for real data analysis because a very long test time is required [12]; (2) ARMA model approach [24] that is model sensitive; (3) PSD approach [25], which can help to identify error parameters but the accuracy of the spectral density estimation has to be considered [26]; and (4) Allan variance approach [27]. In this paper, Allan variance (AVAR) is preferred since it is a time domain analysis technique designed for characterizing noise and stability in clock systems, which can be used to extract the intrinsic noise in the system as a function of the averaging time by analyzing a time sequence [28]. The algorithm and explanation of Allan deviation can be found in [11]. In this paper, twelve hours of static data were collected from the μIMU at room temperature with a sampling frequency of 100 Hz. The outputs were transformed to acceleration in mm/s^2^ and angular rates in °/*s*. The Allan variance plot of the accelerometer and gyroscopes are shown in Figure 3.

According to the theory of Allan variance, different error sources appear on the log-log plot of Allan deviation with different slopes [15]. As for the five stochastic errors in inertial sensor: quantization error, angle/velocity random walk (ARW/VRW), bias instability, rate random walk and rate ramp noise, they appear on the log-log plot of Allan deviation with slopes of −1, −0.5, 0, +0.5 and +1 respectively [29]. By fitting straight lines with the corresponding slopes, the noise sources can be identified, and their magnitudes determined. The red solid straight lines with slope −0.5 in Figure 3a,b are used to estimate the velocity/angle random walk. By examining these two figures it can be concluded that the velocity/angle random walk is dominant at smaller averaging times. When the averaging time increases, the bias instability becomes dominant, which is the minimum point on the curve. When the averaging time increases further, the slope of the curve is not the typical +0.5 or +1, so the rate random walk and rate ramp cannot be extracted directly from the curve. Besides, their influences on pose estimation are not as big as ARW/VRW and bias instability, so we focus on modeling the latter two in this paper. The estimated ARW/VRW walk and bias instability magnitudes are listed in Tables 1 and 2.

As for bias instability, it is modeled as a first-order Gauss-Markov process [11]. Because Gauss-Markov models fits a large number of physical processes with reasonable accuracy, and it has a relatively simple mathematical description. As for velocity/angle random walk, it can be modeled as white noise. Knowing the characteristics of these errors, we need to build a model that is able to predict how these errors vary with time, and apply the model to correct tracking accuracy. Error modeling of these errors will be introduced in Section 2.4.

### 2.3. Rotation Calibration between the Model Coordinate Frame and the World Coordinate Frame

The vision sensor is only capable of estimating its position and orientation (pose) relative to the model coordinate frame. If the model coordinate frame is not aligned accurately with the world coordinate frame, the accelerations could get projected onto the world axes incorrectly. This will induce two problems. Firstly is that the accelerations will be integrated in wrong directions. Secondly acceleration due to gravity cannot be removed correctly. Hence, the relative rotation between model coordinate frame and world coordinate frame should also be calibrated. There are three preconditions for this calibration: (1) the inertial sensor should have been calibrated precisely; (2) the vision sensor should have been calibrated precisely; and (3) the relative rotation ( qIC) between the inertial sensor and the vision sensor should also have been calibrated. The matter of inertial sensor calibration has been discussed in Section 2.2. As for camera calibration, we utilize the Camera Calibration Toolbox for Matlab [30] by taking 15 images with a 9 × 6 checkerboard. And as for the relative rotation ( qIC) calibration between inertial sensor frame and camera frame, the algorithm described in [7] is adopted, which seeks to solve the problem by finding the quaternion that maximizes:

(8) ∑i=1n(qIc⨂aiI⨂q*Ic)⋅vic

where aiI=(0,xiI,yiI,ziI)T represents the measurements of vertical (*g*) by inertial sensor; $v_{i}^{c}={\left(0,x_{i}^{c},y_{i}^{c},z_{i}^{c}\right)}^{T}$ represents a measurement of vertical derived from a selection of vertical vanishing points by vision sensor. These are both represented in quaternion form. Rearranging Equation (8):

(9) (qIc)T(∑i=1n(ViI)TVic)qIc

where *V^I^* and *V^C^* represent measurements by the vertical inertial sensor and the vision sensor. These can be expressed in matrix form as:

(10) $$N=\sum_{i=1}^{n}{\left(V_{i}^{I}\right)}^{T}V_{i}^{c}$$

**Figure 4.**
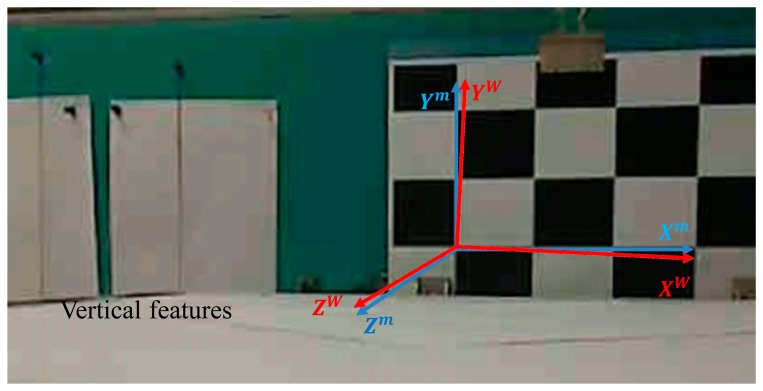
Aligning the world coordinate frame with the model coordinate frame.

The solution to the problem lies in the vector corresponding to the largest eigenvalue of ***N*** [31]. The acquisition of vertical vanishing points is illustrated in Figure 4. The two vertical lines appear parallel in the figure but, actually, they are intersecting at some point outside of the figure. This is the vanishing point we are looking for. Meanwhile, the orientation of vision sensor relative to model frame ( qmc) can also be obtained using the same configuration.

Suppose aII is the gravitational acceleration measured by the accelerometer expressed in quaternion from. Knowing the relative rotation qIc between inertial sensor body frame and camera frame, we may derive its corresponding value in the camera coordinate frame of reference by using the following equation

(11) aIC=qIC⊗aII⊗q*IC

The gravitational acceleration can be further rotated to model coordinate frame by using the orientation quaternion qmc:

(12) aIm=q*mc⊗aIC⊗qmC=q*mc⊗qIC⊗aII⊗q*Ic⊗qmC

where qmc represents the relative rotation from the model frame to the camera frame, as measured by vision sensor by taking the advantage of corner features (see Figure 4).

The true gravitational acceleration in world coordinate frame can be expressed in quaternion form as:

(13) agW=(0,0,−g,0)T

Let qmw=(a,b,c,d)T be a unit quaternion that rotates measurements from the model coordinate frame to the world coordinate frame. The gravitational acceleration can be rotated to model coordinate frame by using the following equation:

(14) agm=q*mw⊗agW⊗qmW

Since the accelerometer has been calibrated accurately, the measured accelerations should be equal to the real gravitational acceleration, that is, aIm=agm. From Equations (12) and (14), the following equation can be obtained:

(15) aIm=q*mw⊗agw⊗qmW

Substituting the values into Equation (15), four nonlinear equations can be obtained:

(16) [2bd−2ad−2bc−a2+b2−c2+d22ab−2cd]g=aIm

Solving the nonlinear equations in Equation (16), the four elements in qmw can be determined.

### 2.4. Error Modeling of a μIC System

#### 2.4.1. Measurement Model

Following the Allan variance analysis, it was found that the inertial measurements in our experiments were corrupted with ARW/VRW and bias instability. Therefore the inertial measurements were modeled as:

(17) $${\tilde{\omega }}_{I}(t)=\omega_{I}(t)+b_{g}(t)+w_{g}(t)$$

(18) $${\tilde{a}}_{I}(t)=a_{I}(t)+b_{a}(t)+w_{a}(t)$$

where *ω_I_*(*t*) and *a_I_*(*t*) represent true angular rates and accelerations; *W_g_*(*t*) and *W_a_*(*t*) represent white Gaussian noises (*E*(*w*) = 0, *E*(*ww^T^*) = σ^2^), which contribute to angle/velocity random walk [32]; and *b_g_*(*t*) and *b_a_* represent the bias instabilities in the gyroscope and the accelerometer respectively that can be modeled as first-order Gauss-Markov processes:

(19) $${\dot{b}}_{g}(\text{t})=-\text{diag}(1./T_{gc})b_{g}(\text{t})+w_{gb}(\text{t})$$

(20) $${\dot{b}}_{a}(\text{t})=-\text{diag}(1./T_{ac})b_{a}(\text{t})+w_{ab}(\text{t})$$

where *T_gc_* and *T_ac_* represent the vectors composed of correlation times of the signals (see Tables 1 and 2); *w_gb_*(t) and *w_ab_* represent white Gaussian noises [12].

#### 2.4.2. State Space Formulation

From Equations (17) and (18), measurement errors in the angular rates and accelerations can be expressed as follows:

(21) $$\delta \omega_{I}(\text{t})=\omega_{I}(t)-\tilde{\omega }(t)=-b_{g}(t)-w_{g}(t)$$

(22) $$\delta a_{I}(t)=a_{I}(t)-{\tilde{a}}_{b}(t)=-b_{a}(t)-w_{a}(t)$$

The error equations for velocity in world coordinate frame are obtained by perturbing the velocity equation [16]:

(23) $$\delta {\dot{v}}_{w}(\text{t})=a_{w}(\text{t})-{\tilde{a}}_{w}(\text{t})=-C_{I}^{w}b_{a}(\text{t})-w_{a}(\text{t})$$

Next the position derivative error ar obtained from the well-known equation:

(24) $$\delta {\dot{p}}_{w}=v_{w}-{\tilde{v}}_{w}=\delta v_{w}$$

The augmented angular rate ***ω̅****_c_* = (0, *ω_x_*, *ω_y_*, *ω_z_*)*^T^* and the quaternion derivative *q̇* is related by:

(25) $$\dot{q}=\frac{1}{2}q⊗{\bar{\omega }}_{c}$$

where *q* = (*q*_1_, *q*_2_, *q*_3_, *q*_4_)*^T^* represents the rotation quaternion that rotates measurements from the camera frame to the world frame [33]. From the properties of quaternion multiplication, Equation (25) can be expressed in matrix form:

(26) $$\dot{q}=\Omega q$$

where:

(27) $$\Omega =\frac{1}{2}\left[\begin{array}{cccc} 0 & -\omega_{x} & -\omega_{y} & -\omega_{z}\\ \omega_{x} & 0 & \omega_{z} & -\omega_{y}\\ \omega_{y} & -\omega_{z} & 0 & \omega_{x}\\ \omega_{z} & \omega_{y} & -\omega_{x} & 0 \end{array}\right]$$

Substituting Equation (21) into Equation (26), the estimated quaternion derivative becomes

(28) $$\dot{\hat{q}}=\tilde{\Omega }\hat{q}=\Omega \hat{q}+B_{g}C_{I}^{c}(-b_{g}-w_{g})$$

where:

(29) $$B_{g}=\frac{1}{2}\left[\begin{array}{ccc} q_{2} & q_{3} & q_{4}\\ -q_{1} & q_{4} & -q_{3}\\ -q_{4} & -q_{1} & q_{2}\\ q_{3} & -q_{2} & -q_{1} \end{array}\right]$$

The error in the quaternion derivative turns out to be:

(30) $$\delta \dot{q}=\dot{q}-\dot{\hat{q}}=\Omega \delta \text{q}+BgC_{I}^{c}b_{g}+B_{g}C_{I}^{c}w_{g}$$

Therefore, these continuous equations can be expressed as follows:

(31) $$\delta \dot{x}(t)=\left[\begin{array}{c} \delta {\dot{p}}_{w}\\ \delta {\dot{v}}_{w}\\ {\dot{b}}_{a}\\ \delta \dot{q}\\ {\dot{b}}_{g} \end{array}\right]=F(t)\delta x(t)+G(t)w(t)$$

where:

(32) $$F(t)=\left[\begin{array}{ccccc} 0 & I & 0 & 0 & 0\\ 0 & 0 & -C(q)C_{I}^{c} & 0 & 0\\ 0 & 0 & -diag(1./T_{ac}) & 0 & 0\\ 0 & 0 & 0 & \Omega & B_{g}\cdot C_{I}^{c}\\ 0 & 0 & 0 & 0 & -diag(1./T_{gc}) \end{array}\right]$$

(33) $$G(t)=\left[\begin{array}{cccc} 0 & 0 & 0 & 0\\ -I & 0 & 0 & 0\\ 0 & I & 0 & 0\\ 0 & 0 & B_{g}\cdot C_{I}^{c} & 0\\ 0 & 0 & 0 & I \end{array}\right]$$

(34) $$w(t)=\left[\begin{array}{c} w_{a}\\ w_{ab}\\ w_{g}\\ w_{gb} \end{array}\right]$$

Discretizing the continuous state space model, and combining the characteristics of the Gauss-Markov process, the discrete state space model can be expressed as follows

(35) $$\delta x\_(k)=A(k)\cdot \delta x(k-1)+w_{d}$$

where:

(36) $$A(k)\approx I+F(t)dt=\left[\begin{array}{ccccc} I & I\cdot dt & 0 & 0 & 0\\ 0 & I & -C(q)\cdot C_{I}^{c}dt & 0 & 0\\ 0 & 0 & diag(\exp(-dt./T_{ac}) & 0 & 0\\ 0 & 0 & 0 & I+\Omega dt & B_{g}\cdot C_{I}^{c}\cdot dt\\ 0 & 0 & 0 & 0 & diag(\exp(-dt./T_{gc}) \end{array}\right]$$

where *dt* is the sampling interval. The discrete error covariance is

(37) $$P\_(k)\approx A(k)P(k-1)A^{T}(k)+G(k)Q(k)G^{T}(k)$$

where the noise covariance matrix *Q* (*k*) is

(38) Q(k)=diag([VRWBIaARWBIg])

and:

(39) $$\begin{array}{c} \text{BIa}=(I-\exp(-2dt./T_{ac}))\cdot \sigma_{\text{BIa}}^{2}\\ \text{BIg}=(I-\exp(-2dt./T_{gc}))\cdot \sigma_{\text{BIg}}^{2} \end{array}$$

#### 2.4.3. Measurement Update

In updating the error state with a set of measurements, it is necessary to know how the measurements vary with time. There are two measurements in our system: the pose of the system as measured by the vision sensor, and the accelerations and angular rates measured by the inertial sensor. Each measurement is defined as the measured pose *p̃* minus the estimated pose *p̂* where *p̂* is pose vector obtained by integrating accelerations and angular rates.

Therefore, the measurement residual can be expressed as:

(40) $$y(k)=(\tilde{p}(k)-\hat{p}(k))-H(k)\delta x\_(k)$$

where *H* (*k*)represents the measurement matrix, and can be expressed as follows

(41) $$H(k)=\left[\begin{array}{ccccc} I_{3\times 3} & 0_{3\times 3} & 0_{3\times 3} & 0_{3\times 4} & 0_{3\times 3}\\ 0_{4\times 3} & 0_{4\times 3} & 0_{4\times 3} & I_{4\times 4} & 0_{4\times 3} \end{array}\right]$$

The state is then updated using the following equations:

(42) $$K(k)=P\_(k)H^{T}(k){\left(H(k)P\_(k)H^{T}(k)+R(k)\right)}^{-1}$$

(43) x(k)=x_(k)+K(k)y(k)

(44) P(k)=(I−K(k)H(k))P_(k)

where *R*(*k*) is measurement noise covariance values determined from experience. Each time the measurement is updated, the pose is corrected using the following equation:

(45) $$p_{c}=\hat{p}+\delta p$$

## 3. Results and Discussion

### 3.1. Trajectory Reconstruction with Proposed Algorithms

**Figure 5.**
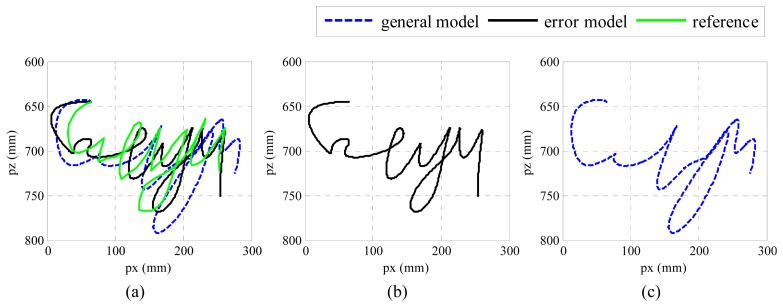
(**a**) Reconstructed “*cityu*” by using general model and error model; (**b**) Reconstructed trajectory using the proposed error model; (**c**) Reconstructed trajectory using the general model.

We performed the experiments by writing five English characters “*cityu*” in one stroke with the μIC system on the platform shown in Figure 1. A pen was attached to one side of the device, so the trajectory of the movement could be recorded on normal white paper. We analyzed the results by comparing the reconstructed trajectory with the one in [33], which we call it general model. In the general model, it assumes that the only noise in inertial sensor is white noise, so the other noises are not modeled. In addition, the deterministic errors in inertial sensor are calibrated by using six-position method, which is not as elegant as Gauss-Newton's method. The comparison is plotted in Figure 5.

From Figure 5c, it can be found the shape of the trajectories can be recovered, although some details may have been lost. Moreover, the estimation position along the *x* direction is stretched a little bit. In Figure 5b, more detailed information has been recovered, and the result is closer to the reference trajectory in both the *x* and *y* directions, which proves that the proposed algorithm may help to recover more detail information.

### 3.2. Experimental Results Evaluation

**Figure 6.**
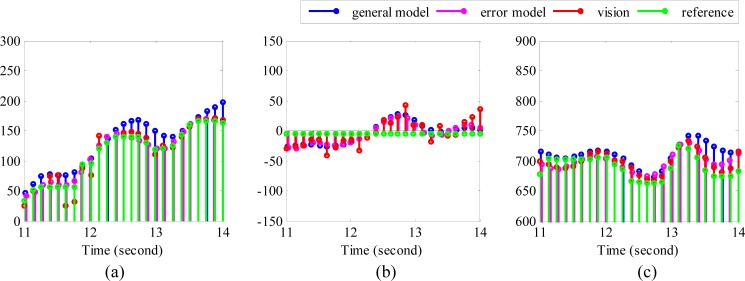
Position alignment *versus* time. (**a**) Position (mm) in *x* direction *versus* time; (**b**) Position (mm) along the *y* direction *versus* time; (**c**) Position (mm) along the *z* direction *versus* time.

With a view to evaluating the performance of the algorithms quantitatively, the RMSE (root mean square error), and the absolute errors associated with each reconstructed trajectory were calculated. The RMSE may give us an overall error level of the reconstructed trajectory. It indicates the overall deviation of the reconstructed trajectory form the real trajectory, while the absolute error may show us the error level of each data point. The calculations however required the temporal information of the reference to be known. Since temporal information cannot be obtained directly from the recorded trajectory, DTW (dynamic time warping) was applied first to align the reference positions with the positions detected by the vision sensor. Next the corresponding temporal information of the reference was estimated. Before performing DTW, the sampling rate of vision sensor was increased from about 5 fps to about 15 fps through interpolation, so that there will be more data points included while calculating RMSE values. The resulting data points used for calculating RMSE are plotted in Figure 6.

**Figure 7.**
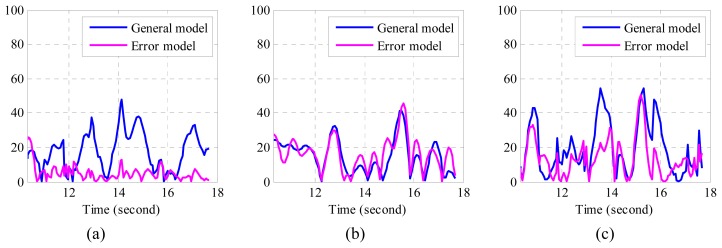
(**a**) Absolute position (mm) error in the *x* direction; (**b**) Absolute position error (mm) in the *y* direction; (**c**) Absolute position error (mm) in the *z* direction.

For absolute error calculation, the following formula was applied:

(46) $$e_{i}=\left|{\hat{x}}_{i}-x_{i}\right|$$

where *x_i_* represents reference position; *x̂_i_* represents estimated position. The corresponding absolute errors obtained by using the two algorithms are plotted in Figure 7.

The RMSE values along each axis were calculated by:

(47) $$\text{RMSE}(\hat{\text{x}})=\sqrt{\frac{\sum_{i=1}^{n}{\left({\hat{x}}_{i}-x_{\text{i}}\right)}^{2}}{n}}$$

where *n* represents the number of measurements. The RMSE values of estimated positions in each axis are listed in Table 3.

From Figure 7, it can be observed that the absolute errors determined by the error model are much smaller than those determined by the general model, especially in *x* direction. From Table 3, we find that the maximum RMSE by employing the error model was 18.38 mm, whereas the maximum RMSE by using the general model was 25.84 mm. That is, the mean RMSE has been reduced by 6.75 mm, while the total RMSE in three directions has been reduced by 20.17 mm when the proposed error model is applied. It is easy to understand if we combine these numbers with the trajectories in Figure 5. Smaller error means the algorithm is able to recover more detailed information of real movement, while the bigger one indicates its distance from real movement. Form both number analysis and trajectory comparison, it can be concluded that the proposed algorithms may help to recover more detailed information, which is usually very important in motion tracking. As a matter of fact, the proposed algorithm is not only confined to application regarding inertial sensor and vision sensor fusion, but also can be applied in the other applications that involve inertial sensors.

## 4. Conclusions

In this paper, algorithms for the μIC system calibration, noise determination and modeling have been presented. For relative rotation calibrations between the two coordinate frames, (inertial sensor body coordinate frame and camera coordinate frame, and model coordinate frame and world coordinate frame), we have shown that only one reference image is required. For deterministic error calibration of the inertial sensor, Gauss-Newton's method has been adopted to iteratively refine the calibration results. We demonstrated that as long as the parameters are chosen properly, this algorithm is able to yield results with given precision in seconds. We then modelled the stochastic errors identified in the inertial sensor, and applied the proposed noise model to the inertial sensor and vision sensor fusion. By comparing the results with and without error correction, we found that the RMSE of reconstructed hand-writing trajectories with error correction have been reduced by 6.75 mm over a 30 cm by 15 cm writing area. Moreover, we have also successfully applied DTW for temporal information retrieval.

Although good results have been achieved, there is still much work to be done in realizing a mobile motion tracking and recognition system based on the fusion of MEMS motion sensors and vision sensors. For example, the radial distortion of the web-based camera should be resolved. As can be seen from Figure 5a, the trajectory in *x* direction, which is the radial direction of the camera, has been “stretched”. This type d of distortion will deteriorate the fusion results, and hence, a more reliable distortion correction model should be constructed. Another example is the “image blurring” during fast movement of an input motion. Although the pose could be estimated by using inertial senor during the absence of vision sensor, the pose estimation results by using only inertial sensor will deteriorate quickly when the images are lost too often.

## Figures and Tables

**Table 1.** Bias instability and velocity random walk of the accelerometer.

	**Bias Instability**	**Velocity Random Walk**
**X axis**	9.086 ± 0.201 mm/s^2^ (at 10.24 s)	18.946 ± 0.419 mm/s^2^/ $\sqrt{s}$
**Y axis**	8.540 ± 0.189 mm/s^2^ (at 10.24 s)	18.154 ± 0.401 mm/s^2^/ $\sqrt{s}$
**Z axis**	5.560 ± 0.174 mm/s^2^ (at 20.48 s)	17.520 ± 0.548 mm/s^2^/ $\sqrt{s}$

**Table 2.** Bias instability and angle random walk magnitudes associated with the gyroscopes.

	**Bias Instability**	**Angle Random Walk**
**X axis**	0.03287 ± 0.000073 °/s (at 10.24 s)	0.058479 ± 0.001293 °/ $\sqrt{s}$
**Y axis**	0.021762 ± 0.005816 °/s (at 1310.72 s)	0.066069 ± 0.017658 °/ $\sqrt{s}$
**Z axis**	0.032553 ± 0.001018 °/s (at 20.48 s)	0.064275 ± 0.002011 °/ $\sqrt{s}$

**Table 3.** The root mean square error (RMSE) values associated with the estimated positions.

**Algorithm**	**X (mm)**	**Y (mm)**	**Z (mm)**	**Mean (mm)**	**Total (mm)**
**General model**	20.47	17.6	25.84	21.33	63.91
**Error model**	7.47	17.89	18.38	14.58	43.74

## References

[1] Shin D.H., Jang W.S. (2009). Utilization of Ubiquitous Computing for Construction AR Technology. Autom. Constr..

[2] Welch G., Foxlin E. (2002). Motion Tracking: No Silver Bullet, but a Respectable Arsenal. IEEE Comput. Graph. Appl..

[3] Zhou S., Shan Q., Fei F., Li W.J., Kwong C.P., Wu P.C.K., Meng B., Chan C.K.H., Liou J.Y.J. Gesture Recognition for Interactive Controllers Using MEMS Motion Sensors.

[4] Aron M., Simon G., Berger M.O. (2007). Use of Inertial Sensors to Support Video Tracking. Comput. Animat. Virtual Worlds.

[5] Saeedi P., Lawrence P.D., Lowe D.G. (2006). Vision-Based 3D Trajectory Tracking for Unknown Environments. IEEE Trans. Robot..

[6] Dias J., Vinzce M., Corke P., Lobo J. (2007). Editorial Special Issue: 2nd Workshop on Integration of Vision and Inertial Sensors. Int. J. Robot. Res..

[7] Lobo J., Dias J. (2007). Relative Pose Calibration between Visual and Inertial Sensors. Int. J. Robot. Res..

[8] Bishop G., Welch G. An Introduction to the Kalman Filter.

[9] Syed Z., Aggarwal P., Goodall C., Niu X., El-Sheimy N. (2007). A New Multi-Position Calibration Method for MEMS Inertial Navigation Systems. Meas. Sci. Technol..

[10] Frosio I., Pedersini F., Borghese N.A. (2009). Autocalibration of MEMS Accelerometers. IEEE Trans. Instrum. Meas..

[11] Petkov P., Slavov T. (2010). Stochastic Modeling of MEMS Inertial Sensors. Cybern. Inf. Technol..

[12] Saini V., Rana S., Kuber M. (2010). Online Estimation of State Space Error Model for MEMS IMU. J. Model. Simul. Syst..

[13] Brown R.G., Hwang P.Y. (1992). Introduction to Random Signals and Applied Kalman Filtering.

[14] Lee J., Hong S., Moon N., Oh S.-J. (2010). Acoustic Sensor-Based Multiple Object Tracking with Visual Information Association. EURASIP J. Adv. Signal. Process..

[15] El-Sheimy N., Hou H., Niu X. (2008). Analysis and Modeling of Inertial Sensors Using Allan Variance. IEEE Trans. Instrum. Meas..

[16] Friedland B. (1978). Analysis Strapdown Navigation Using Quaternions. IEEE Trans. Aerosp. Electron. Syst..

[17] Chroust S., Vincze M. (2004). Fusion of Vision and Inertial Data for Motion and Structure Estimation. J. Robot. Syst..

[18] Armesto L., Tornero J., Vincze M. (2007). Fast Ego-Motion Estimation with Multi-Rate Fusion of Inertial and Vision. Int. J. Robot. Res..

[19] Armesto L., Tornero J., Vincze M. (2008). On Multi-Rate Fusion for Non-Linear Sampled-Data Systems: Application to a 6D Tracking System. Robot. Auton. Syst..

[20] Hol J.D., Schön T.B., Luinge H., Slycke P.J., Gustafsson F. (2007). Robust Real-Time Tracking by Fusing Measurements from Inertial and Vision Sensors. J. Real-Time Image Process..

[21] Yi Y. (2007). On Improving the Accuracy and Reliability of GPS/INS-Based Direct Sensor Georeferencing.

[22] Lu C.-P., Hager G.D., Mjolsness E. (2000). Fast and Globally Convergent Pose Estimation from Video Images. IEEE Trans. Pattern Anal. Mach. Intell..

[23] Titterton D., Weston J., Zarchan P. (2004). Strapdown Inertial Navigation Technology.

[24] Seong S.M., Lee J.G., Park C.G. (2000). Equivalent Arma Model Representation for RLG Random Errors. IEEE Trans. Aerosp. Electron. Syst..

[25] (1999). IEEE Standard Specification Format Guide and Test Procedure for Linear, Single-Axis, Non-Gyroscopic Accelerometers. IEEE Std 1293–1998.

[26] Quinchia A.G., Ferrer C., Falco G., Falletti E., Dovis F. Analysis and Modelling of MEMS Inertial Measurement Unit.

[27] (1996). IEEE Standard Specification Format Guide and Test Procedure for Single-Axis Laser Gyros. IEEE Std 647–1995.

[28] Niu X., Chen Q., Zhang Q., Zhang H., Niu J., Chen K., Shi C., Liu J. (2013). Using Allan Variance to Analyze the Error Characteristics of GNSS Positioning. GPS Solut..

[29] Tehrani M. Ring Laser Gyro Data Analysis with Cluster Sampling Technique.

[30] Bouguet J.-Y. Camera Calibration Toolbox for Matlab. http://www.vision.caltech.edu/bouguetj/calib_doc/.

[31] Horn B.K. (1987). Closed-Form Solution of Absolute Orientation Using Unit Quaternions. J. Opt. Soc. Am..

[32] Woodman O.J. (2007). An Introduction to Inertial Navigation.

[33] Zhou S., Fei F., Zhang G., Mai J.D., Liu Y., Liou J.Y.J., Li W.J. (2014). 2D Human Gesture Tracking and Recognition by the Fusion of MEMS Inertial and Vision Sensors. IEEE Sens. J..

